# Evolutionary Interactions between N-Linked Glycosylation Sites in the HIV-1 Envelope

**DOI:** 10.1371/journal.pcbi.0030011

**Published:** 2007-01-19

**Authors:** Art F. Y Poon, Fraser I Lewis, Sergei L. Kosakovsky Pond, Simon D. W Frost

**Affiliations:** 1 Department of Pathology, University of California San Diego, La Jolla, California, United States of America; 2 Institute of Evolutionary Biology, University of Edinburgh, Edinburgh, Scotland, United Kingdom; ETH Zürich, Switzerland

## Abstract

The addition of asparagine (N)-linked polysaccharide chains (i.e., glycans) to the gp120 and gp41 glycoproteins of human immunodeficiency virus type 1 (HIV-1) envelope is not only required for correct protein folding, but also may provide protection against neutralizing antibodies as a “glycan shield.” As a result, strong host-specific selection is frequently associated with codon positions where nonsynonymous substitutions can create or disrupt potential N-linked glycosylation sites (PNGSs). Moreover, empirical data suggest that the individual contribution of PNGSs to the neutralization sensitivity or infectivity of HIV-1 may be critically dependent on the presence or absence of other PNGSs in the envelope sequence. Here we evaluate how glycan–glycan interactions have shaped the evolution of HIV-1 envelope sequences by analyzing the distribution of PNGSs in a large-sequence alignment. Using a “covarion”-type phylogenetic model, we find that the rates at which individual PNGSs are gained or lost vary significantly over time, suggesting that the selective advantage of having a PNGS may depend on the presence or absence of other PNGSs in the sequence. Consequently, we identify specific interactions between PNGSs in the alignment using a new paired-character phylogenetic model of evolution, and a Bayesian graphical model. Despite the fundamental differences between these two methods, several interactions are jointly identified by both. Mapping these interactions onto a structural model of HIV-1 gp120 reveals that negative (exclusive) interactions occur significantly more often between colocalized glycans, while positive (inclusive) interactions are restricted to more distant glycans. Our results imply that the adaptive repertoire of alternative configurations in the HIV-1 glycan shield is limited by functional interactions between the N-linked glycans. This represents a potential vulnerability of rapidly evolving HIV-1 populations that may provide useful glycan-based targets for neutralizing antibodies.

## Author Summary

Many viruses exploit the complex machinery of the host cell to modify their own proteins, by the enzymatic addition of sugar molecules to specific amino acids. These sugars, or “glycans,” play several important roles in the infective cycle of the virus. The envelope of the human immunodeficiency virus type 1 (HIV-1), for example, becomes coated with so many glycans that the virus can become invisible to the protein-specific immune response of the host. Although some glycans are evolutionarily conserved, many others may be present within some hosts but absent in others, and may even appear or disappear over the course of an infection in a single host. To understand this variability, we have analyzed HIV-1 envelope sequences to identify cases where the presence of one glycan was dependent on the presence or absence of another (called glycan–glycan interactions). We used two newly developed computational methods to detect these interactions, thereby providing conclusive evidence of a new fundamental pattern: the glycans that exclude each other tend to occur near the same spot on the envelope, whereas glycans that occur together tend to be far apart.

## Introduction

Proteins are frequently modified during or after translation by the enzymatic attachment of polysaccharide chains (i.e., glycans) to amino acid residues. The addition of glycans to asparagine residues is known as N-linked glycosylation and occurs widely in eukaryotes and archaebacteria, but only rarely in prokaryotes [1]. N-linked glycosylation targets an amino acid sequence motif that is defined by NX_1_(S/T)X_2_, where X represents any amino acid other than proline [2]. Glycosylation by the host cell can strongly influence the folding, stability, and biological function of virus-encoded proteins [3–5]. As a result, many viral sequences contain a large number of conserved potential N-linked glycosylation sites (PNGSs) [6,7].

For instance, the surface glycoprotein (gp120) of the human immunodeficiency virus type 1 (HIV-1) envelope, which represents the primary interface between the virus and the host environment, is one of the most heavily glycosylated proteins known to date, with nearly half of its molecular weight due to the addition of N-linked glycans [8]. The transmembrane glycoprotein (gp41) of the HIV-1 envelope is also glycosylated, but to a lesser extent. The addition of N-linked glycans is essential for HIV-1 gp120 to fold into the proper conformation to bind to the CD4 receptor [9], and influences the binding of alternative coreceptors [7,10], the combined effects mediating the fusion and entry of HIV-1 into the host cell. Furthermore, N-linked glycans can sterically prevent neutralizing antibodies from accessing the antigenic polypeptide surface of the HIV-1 envelope, thereby protecting the virus from the humoral immune response with a “glycan shield” [11]. Strains of HIV-1 in which N-linked glycosylation sites have been experimentally deleted or modified can become more sensitive to neutralization [12]. However, the relative contribution of the glycan shield to protection from neutralization in vivo remains unresolved [13].

Because many N-linked glycans are highly conserved components of the HIV-1 envelope, they may themselves provide a promising target for neutralizing antibodies; for instance, the broadly neutralizing human monoclonal antibody 2G12 binds to an epitope comprising N-linked glycans that are attached to the gp120 glycoprotein [14]. There is, however, abundant evidence that host-specific adaptation of HIV-1 can involve the mutational gain or loss of PNGSs. For example, codon positions under strong host-specific selection in HIV-1 gp120 sequences often occur within PNGSs [11,15,16]. Although many PNGSs are evolutionarily conserved, there is nonetheless ample variation in the distribution of PNGSs among HIV-1 sequences that is poorly understood. Moreover, empirical evidence suggests that the influence of PNGSs on the fitness of HIV-1 is context-dependent, i.e*.*, conditional on the presence or absence of other PNGSs in the viral sequence. First, the experimental reintroduction of individual PNGSs to a neutralization-sensitive HIV-1 clone by Wei et al. [11] had only modest effects on neutralization sensitivity, yet the combined introduction of multiple PNGSs revealed a positive interaction causing a disproportionate increase in resistance to neutralization. Second, Ohgimoto et al. [17] demonstrated that a strain of simian immunodeficiency virus (SIV) could not tolerate the removal of multiple N-linked glycans from gp120, if the glycans were specifically removed from the same location. Apart from these findings, the extent and identity of such interactions among PNGSs that constitute the HIV-1 glycan shield has not yet been systematically characterized.

### Detecting Interactions

Developing an efficient and accurate method to detect interactions between components of the same protein, or between different proteins, is an important and unresolved problem in computational biology. One of the first approaches was to apply a measure of the correlation in amino acid composition at different codon positions in related protein sequences [18,19]. However, this approach was limited to pairwise interactions and could not readily distinguish functional from phylogenetic relationships [20]. A complete representation of interactions in a phylogenetic context can be achieved by modeling the evolution of the entire sequence through a state space of all possible sequences [21,22]. This approach involves an excessive number of parameters—i.e., on the order of *O*(20*^L^*) parameters for protein sequences of length *L*—requiring approximate methods of parameter estimation that are not necessarily statistically robust (e.g., Markov Chain Monte Carlo, MCMC). Another class of phylogenetic models include the simplifying assumption that the overall rate of evolution at a site depends on the states of adjacent sites [23,24], contrary to the intuition that an interaction drives substitutions to specific states. Even so, these models remain computationally inefficient.

In sum, there are a number of diverse methods that can be applied towards identifying nonindependent evolution between positions in a sequence, but no consensus on which method performs most reliably in practice. Furthermore, other potentially useful approaches (e.g., Bayesian networks) that can address specific weaknesses of existing methods have yet to be applied to the study of covariation within evolving sequences. We propose to employ new methods that complement each other's strengths, while addressing the weaknesses of previous methods, to determine the subset of detectable interactions that are robust to varying assumptions.

In this study, we evaluate whether the evolution of PNGSs in the HIV-1 envelope has been significantly influenced by glycan–glycan interactions, and identify specific interactions between PNGSs in the envelope glycoproteins gp120 and gp41 by the application of phylogenetic and graphical models to a published alignment of HIV-1 sequences. First, we verify that the distribution of PNGSs is consistent with heterotachy, in which site-specific rates of evolution vary over time due to genetic or environmental interactions [25]. This is accomplished by evaluating a hidden rate-switching or “covarion” model [26,27] against constrained phylogenetic models of PNGS evolution in a nested analysis based on maximum likelihood. Second, we propose a stochastic model to directly identify genetic interactions, using a new paired-character model of PNGS evolution named the *disequon* model (where *sequon* refers to the sequence motif defining a PNGS). We use maximum likelihood estimates of the disequon model parameters to quantify the type and magnitude of interactions between every pairwise combination of PNGSs in the alignment, within a phylogenetic context. However, because each pairwise interaction is evaluated in isolation from all other PNGSs, the model is unable to identify higher-order interactions among PNGSs, and therefore cannot guarantee that specific pairwise interactions persist in the context of the entire glycan shield.

To capture these higher-order interactions, we investigate interactions among PNGS using a probabilistic graphical model. A graph consists of a number of nodes, each one representing a variable (e.g., presence/absence of a PNGS), that are connected by directed or undirected arcs. The conditional dependence of one variable upon another is represented by a directed arc connecting the corresponding nodes, usually depicted as an arrow. Nodes that are not connected by arcs are conditionally independent. In practice, a directed graph is required to be acyclic so that a chain of directed arcs does not form a feedback loop to its parent node—such a graph is commonly referred to as a tree or “forest” of unrelated trees. Probabilistic models based on directed acyclic graphs originate from the formulation of path analysis by the evolutionary biologist Sewall Wright in 1921 [28], but are currently more widely known as Bayesian networks [29]. There is an ongoing proliferation of studies that apply Bayesian networks to problems in biology, such as the analysis of gene expression data to infer the structure of regulatory networks [30]. Bayesian networks confer several advantages for analyzing biological data: (i) networks provide an intuitive visual representation of biological complexity; (ii) they can explicitly incorporate experimental uncertainty and missing observations; and foremost, (iii) an abundance of algorithms for the inference of Bayesian networks from empirical data is already available (reviewed in [31]).

## Results

### Diversity of PNGSs in HIV-1 Glycoproteins

We located 224 positions at which PNGSs occurred in at least one sequence, out of 1,049 possible positions in an alignment of 711 full-length HIV-1 envelope sequences. The frequency of PNGS per codon position was highly variable, with more than 65% of PNGSs present in fewer than 10% of the sequences. About one-quarter of PNGSs (*n* = 57) were unique to a single sequence in the alignment and represented either spurious variation in the position of a PNGS due to uncertainty in the alignment of sequences or genuinely rare glycosylation sites. An individual sequence encoding both gp120 and gp41 contained 29.9 PNGS on average. We fitted a generalized linear model to the number of PNGS per sequence (using the *R* function *glm* [32]), which revealed subtype to be a significant factor (Wald test: χ_4_^2^ = 12.7, *p* = 0.012). This analysis was restricted to subtypes that were represented by at least 40 nonrecombinant sequences in the data (viz. A, B, C, D, and O) to avoid spurious results due to small sample size. This effect of subtype was largely due to an elevated number of PNGSs (≈31.9) in O group sequences (*n* = 44; *z* = 2.8, *p* = 0.005). For example, the PNGSs N59 and N229 (numbered according to their positions in the HxB2 reference sequence) were essentially unique to group O sequences, as noted in a previous study [7].

### Covarion Model Analysis

Likelihood and parameter estimates for the covarion model and constrained models of PNGSs evolution are summarized in Table S1. Variation among sites in the overall rate of gain or loss of PNGS (α^−1^) was strongly supported by the data (χ_1_^2^ = 960.4, *p* ≪ 0.001). This result was consistent with prior observations that while some PNGSs are highly conserved in HIV-1 envelope sequences, others are under strong host-specific selection [7,11]. We found strong support for rejecting the noncovarion model in favor of a model with an unconstrained rate of switching between hidden states (*s*_on_, *s*_off_ > 0; χ_2_^2^ = 639.8, *p* ≪ 0.001). Allowing variation among sites in the hidden-state switching rates (σ^−1^) provided an additional significant improvement of fit to the data (χ_1_^2^ = 174.2, *p* ≪ 0.001). Although the mean switching rates (*s*_on_ and *s*_off_) appeared to be roughly symmetric in the full model, the nested model with the constraint *s*_off_ = *s*_on_ was rejected by a likelihood-ratio test (LRT) (χ_1_^2^ = 6.6, *p* = 0.01). In sum, the full covarion model was favored by all criteria evaluated (AIC, AIC*_c_*, and BIC; Table S1) with the following parameter estimates: *s*_on_ = 0.08, *s*_off_ = 0.06, *r*_01_/*r*_10_ = 0.29, σ = 0.75, and α = 0.46. Removal of sequences that were annotated as subtype recombinants (*n* = 219) had no discernible effect on our results. Overall, the hypothesis that evolutionary rates for the gain or loss of PNGSs were variable over time, i.e., heterotachy, was strongly supported by the data.

### Disequon Model Analysis

We detected significant interactions between PNGSs in the disequon model for 22 out of 6,455 pairwise combinations after applying a deliberately conservative Bonferroni correction for multiple comparisons (see Materials and Methods). Nine of these pairs consisted of PNGSs that overlapped by one or three residues and were located either in the variable loops of gp120 (i.e., V1/V2 and V4) or gp41 (i.e., N624–N625). Estimates of the interaction parameter (ɛ) indicated that the interactions between all overlapping pairs were negative (ɛ < 1), with a single exception. This exception involved PNGSs introduced by insertions into the V1/V2 loop (alignment positions 252 and 253) that were present at low frequencies in the alignment (1.1% and 1.4%, respectively). However, both PNGSs occurred together in a single subtype B sequence, resulting in an unusually high estimate of ɛ = 15.3 (χ_1_^2^ = 17.5, *p* = 2.91 × 10^−8^). It is worth noting that an unusually high localized density of PNGSs was introduced into this sequence by the insertion of the motif NNTSNNTSY into the V1/V2 loop, defining four distinct PNGSs. This particular outcome was handled as an outlier in subsequent analyses.

**Figure 1:**
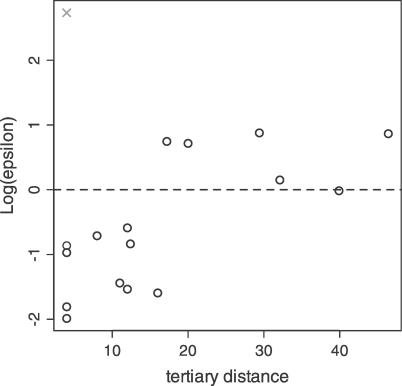
The Nature of Glycan Interactions Depends on Their Intramolecular Distance Potential PNGSs that were identified by the disequon model as having significant interactions with other PNGSs were mapped to a structural model of HIV-1 gp120. Intramolecular distances between interacting PNGSs were measured using the visualization software UCSF Chimera [53]. When an asparagine residue corresponding to a PNGS was not present in the structural model, the distance between pairs was approximated by 4.0 Å times the number of residues separating the PNGSs, to a maximum of five residues. If the number of residues exceeded five, then the unmapped pair was omitted from the analysis.

Table 1 summarizes the parameter estimates from the disequon model for all nonoverlapping pairwise combinations of PNGSs with a significant interaction component (ɛ ≠ 1). Unlike the overlapping PNGSs, positive interactions between nonoverlapping PNGSs (ɛ > 1, *n* = 8) occurred about as often as negative interactions (ɛ < 1, *n* = 5). Indeed, we found that ɛ increased significantly with respect to the intramolecular distance (measured in angstroms, Å), separating the asparagine residues of PNGSs with significant pairwise interactions (linear regression: *F* = 15.8, df = 1, *p* = 0.001; Figure 1). A similar trend was observed between ɛ and the primary distance (i.e., the number of residues separating the PNGSs in the amino acid sequence; *F* = 10.0, df = 1, *p* = 0.005). In other words, positive interactions tended to be restricted to PNGSs that were sufficiently distant from one another in the HIV-1 envelope glycoproteins.

**Table 1:**
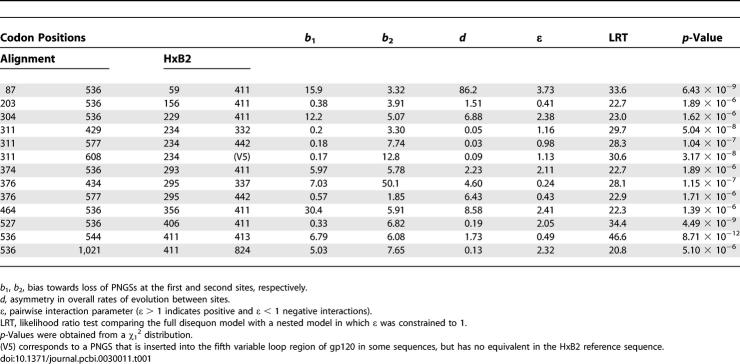
Parameter Estimates for Nonoverlapping PNGSs with Significant Interactions in the Disequon Model

Many pairwise interactions detected by the disequon model involved PNGS that were located in the V4 loop of the HIV-1 envelope protein gp120 (i.e., N406, N411, N413). Also, several PNGSs were involved in multiple pairwise interactions (e.g., N234, N295, and N411). The insertion of the sequon N411 in the V4 loop, for example, was involved in eight of the 13 significant pairwise interactions between nonoverlapping PNGS, which suggested that N411 has a broad influence on formation of a functional glycan shield.

### Bayesian Network Analysis

**Figure 2:**
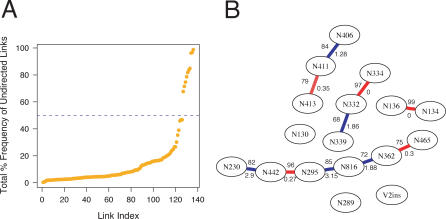
Consensus Bayesian Network of Highly Polymorphic PNGSs in the HIV-1 Envelope (A) Ordered distribution of frequencies that undirected links (i.e., arcs) occurred in replicate networks optimized on 100 random samples of 200 sequences from the alignment, expressed as a percentage (*y*-axis). A dotted line at 50% indicates an arbitrary cutoff, above which arcs were applied to assembling the consensus network. (B) Graph of consensus network. Each node (oval) represents the PNGS identified by its position in the HxB2 reference sequence, with one exception (V2ins) that represents an inserted PNGS downstream of the conserved N186. Arcs between nodes are labeled with percentage of occurrence above and odds-ratios below. An odds-ratio <1 (red) indicates mutual exclusion and >1 (blue) co-occurrence of PNGSs.

To reduce the computational complexity of the analysis while retaining our power to detect higher-order interactions, we restricted our network search to a subset of 17 PNGS that occurred at intermediate frequencies (20%–80%) in the alignment (Table S2). Figure 2A summarizes the frequencies at which arcs connecting two PNGS in either direction occurred in a total of 25,000 Bayesian networks (inferred from 250 replicate optimizations times 100 random samples from the alignment). Arcs between ten specific pairs of PNGS occurred in more than 67% of the networks generated from random samples. The next most frequent undirected arc occurred in only 47% of the networks (Figure 2A). Consequently, we applied the ten arcs with strongest support towards assembling a majority-rule consensus Bayesian network.

Figure 2B illustrates the consensus network assembled from the ten undirected arcs with support values above the threshold, which connected 14 of the 17 PNGSs. Several PNGSs were conditionally dependent on more than one other PNGS in the network, implying higher-order interactions that would not have been captured by the disequon model. The posterior-odds ratios (see Materials and Methods) associated with the consensus arcs N134–N136 and N332–N334 indicated that PNGSs were mutually exclusive at these sites, which in both cases overlapped by two residues in the amino acid sequence. This outcome was hardly surprising because it is impossible for two well-defined PNGSs to overlap by two residues. However, the majority of arcs in the consensus network represented long-range interactions. The most acute instance of this occurred between the PNGSs N295, N362, and N816, in which the latter is located on the cytoplasmic tail of the transmembrane glycoprotein gp41, and the other two are located on the outer surface of gp120.

This network analysis could not account for phylogenetic relationships between sequences. To identify potential effects of phylogeny in the consensus network, we generated networks for subsets of HIV-1 envelope sequences that were annotated as belonging to subtypes A, B, C, and D, or circulating recombinant forms CRF01 and CRF02, using an MCMC-based procedure that was designed for inferring networks from small datasets. Because the number of CRF02 sequences (*n* = 17) was far too small to perform any rigorous network analysis on 17 variables, the results from this network were omitted. Overall, five out of the ten arcs from the consensus network were recovered in more than one subtype-specific network (Table 2). This subset included every interaction that was identified by both the consensus network and the disequon model. The absence of the remaining consensus network interactions was possibly either caused by subtype-founder effects, or represented an artifact of limited sample size within subtypes.

**Table 2:**
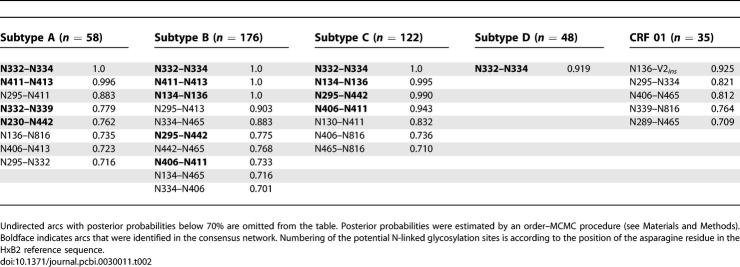
Posterior Probabilities of Undirected Arcs in Subtype-Specific Networks

**Figure 3:**
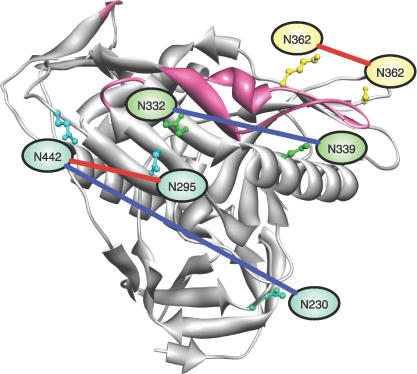
Interacting PNGS Mapped to Structural Model of gp120 Arcs from the consensus network are mapped to asparagine residues in the structure of a folded CD4-bound gp120 protein. The host cell-binding face of gp120 is oriented towards the top of the figure, and variable loops (of which many residues are truncated) are highlighted in pink. Arcs are colored red to indicate mutual exclusion, and blue indicates co-occurrence of the PNGSs. The asparagine residues of mutually exclusive PNGSs N295/N442 and N362/N465 are located within ∼12 Å of one another. In contrast, co-occurent PNGSs are separated by much greater intramolecular distances.

We mapped PNGS from the consensus network to the predicted three-dimensional structure of the folded glycoprotein gp120 (Figure 3). Because several regions of gp120 were truncated in the structural analysis (e.g., variable loop V1/V2), only seven PNGSs from the consensus network (N230, N295, N332, N339, N362, N442, and N465) could be mapped to the structural model [33]. Of the four arcs from the consensus network defined by these PNGSs, two represented positive interactions (N332–N339 and N230–N442) and the remaining two negative interactions (N362–N465 and N295–N442). Only one of these four interactions (N295–N442) had been previously detected by our disequon analysis. Although this was an insufficient sample for a robust comparison, the asparagine residues within PNGSs that participated in negative interactions were located closer together (8.7 Å and 10.8 Å) than those in positive interactions (14.8 Å and 42.0 Å). This trend lent further support for our observation that negative interactions tended to occur between co-localized PNGSs, and positive interactions between distant PNGSs.

## Discussion

### Constraints on the Glycan Shield

Although there is substantial evidence that the distribution of PNGSs in the HIV-1 envelope glycoproteins is a perpetually evolving phenotype, little is known about the complexities of how it responds to host-specific selection. The existence of negative interactions between PNGSs implies that there is more than one way for the glycan shield to adapt to the selective pressures of a given environment. To determine whether this prediction is borne out empirically, we evaluated the extent of convergent evolution of PNGSs in a longitudinal study of 11 macaques that were experimentally infected with a chimeric simian/human immunodeficiency virus strain (SHIV-86P) containing an HIV-1 subtype B–derived envelope coding region, as reported recently by Blay et al. [34]. Despite substantial divergence in the amino acid sequences from the replicate populations, convergent evolution occurred at several PNGSs (N141, N188, N276, N386, N397, and N462), suggesting that the selective response was highly constrained at those positions. In other words, the selective advantage of these changes was apparently independent of the presence or absence of other PNGS in the envelope sequence. We have found no evidence in our study that these PNGSs are involved in any interactions. However, three out of the four PNGS at which highly divergent evolution occurred (N136, N234, and N362) have been implicated by our study in interactions with other PNGSs. Hence, the complexity of the adaptive response of the glycan shield can be predicted by the interactions that we have identified between PNGSs.

Moreover, we have found evidence of a novel association between the distance separating PNGSs and the type of interaction between them, which represents an additional layer of constraints on the glycan shield. Negative, or “mutually exclusive,” interactions tended to occur between PNGSs that occupied similar locations on the glycoprotein. This tendency for overlapping or adjacent PNGS to be mutually exclusive may have been caused by either steric hinderance or functional redundancy. Because of the large molecular weight of N-linked glycans, steric hindrance could conceivably prevent glycans attached to overlapping PNGSs from occupying the same space [2]. However, it is possible for overlapping PNGSs offset by one residue to become simultaneously glycosylated [35]. Functional redundancy, on the other hand, implies that having additional glycans at a given location on the envelope glycoprotein would fail to provide any further protection from neutralization while accruing a debilitating cost to the normal functioning of the glycoprotein [36]. Either mechanism would enforce a maximum limit on the number of PNGSs in the sequence, which could be maximized by evenly distributing PNGSs across the protein surface or functional space.

Positive interactions, on the other hand, tended to occur exclusively between PNGSs that were separated by greater distances. The observed relationship between spatial distance and the interaction value (ɛ) suggested a minimum distance threshold of 20 Å, separating glycans for them to become nonredundant, i.e., to achieve complete coverage of the antigenic surface of gp120 (Figure 1). For comparison, high mannose and complex carbohydrates were estimated to extend from the glycoprotein surface to a distance of approximately 30–40 Å and to have a maximum diameter of about 15–40 Å [37]. Evidence of positive interactions between distant PNGS implies that the glycan shield is a biological gestalt, unable to function until it achieves complete coverage of HIV-1 envelope glycoproteins.

These spatial constraints predicted by our study complements a similar constraint that was previously put forth on the basis of experimental results from Ohgimoto et al. [17], in which the infectivity of a strain of SIV was sensitive to the cumulative depletion of N-linked glycans specifically when they were removed from a similar location on the gp120 glycoprotein. In other words, this experiment revealed a selective advantage for maintaining a minimum local density of glycans. Here we find evidence of similar constraints preventing the local density of glycans from exceeding a maximum threshold, and maintaining a minimum overall density of glycans on the HIV-1 envelope glycoproteins.

### V3 Loop Glycan Interactions

The third variable loop (V3) is an immunologically and functionally important region of the HIV-1 gp120 amino acid sequence (reviewed in [38]). There are several conserved PNGSs located within or adjacent to V3 (N289, N295, N301, N332, and N339), which may function primarily to mask the large number of neutralizing antibody epitopes defined in this region. Additionally, the PNGSs N295 and N332 contribute to the formation of a glycan-dependent epitope that is recognized by the human monoclonal neutralizing antibody 2G12 [14]. In light of these factors, we are particularly interested in describing interactions involving PNGSs that are associated with V3. For instance, our disequon analysis revealed a strong negative interaction between the PNGSs N295 and N337, where the latter replaces N339 in a small number (*n* = 21) of sequences belonging mostly to subtypes B and C. In the presence of N337, the PNGS at N295 was intact in only two sequences, suggesting that N337 may provide an infrequently used alternative that can provide similar protection for epitopes on V3, by shifting the N-linked glycan from N339 closer to the space normally occupied by N295. A second alternative for N295 may also be provided by the PNGS N442, which is also located near the base of V3 in the folded gp120 glycoprotein (Figure 3).

### V4 Loop Glycan Interactions

An intriguing result from our phylogenetic and Bayesian network analyses on the distribution of PNGS in HIV-1 *env* sequences was the strong evidence of interactions involving PNGSs that were located in the V4 loop of gp120 (N406, N411, N413). Previous empirical studies have noted that the modification of PNGSs in V4 was prominent during adaptation of HIV-1 and SIV to a novel host [11,34,39] and that the addition or removal of PNGSs in V4 affected neutralization sensitivity in a context-dependent fashion, involving interactions with PNGSs located elsewhere in gp120 [11]. The V4 loop is an accessible and flexible region in the gp120 structure [33]. Glycans in V4 are apparently nonessential for CD4 binding, and the V4 region has not been associated with any other known biological function of gp120 [40]. Therefore, the V4 region may exist solely to facilitate viral escape as a component of the evolving glycan shield [41].

Using a disequon model, we found significant interactions between N411 and eight other PNGSs in the HIV-1 envelope glycoproteins, of which six were located outside of V4 (Table 1). Six of these eight interactions were positive, suggesting that N411 might represent a central component in a high-density configuration of the glycan shield. Indeed, sequences in which a PNGS was present at N411 contained a significantly greater number of PNGSs in the rest of the sequence (generalized linear model, Wald test: χ_1_^2^ = 8.44, *p* < 0.004). Curiously, one of these positive interactions implicated a PNGS (N824) located on the cytoplasmic tail of the transmembrane envelope glycoprotein gp41. This evidence of a functional interaction between PNGSs situated on the gp120 V4 loop extruding outward and the inner face of gp41 suggests that the glycosylation state can be transmitted between these noncovalently associated glycoproteins of the HIV-1 envelope, perhaps by inducing a cascade of conformational change, which has been postulated for amino acid substitutions and deletions within gp41 in previous studies [42,43].

### Implications for Vaccine Development

The N-linked glycosylation of the HIV-1 envelope glycoproteins is one of the most important protective mechanisms to overcome in order to develop an effective neutralizing antibody-inducing vaccine. Because several N-linked glycosylation sites are relatively constant across HIV-1 subtypes, there is a great deal of interest in developing a carbohydrate-based antigen designed to elicit a humoral immune response to HIV-1. However, even the N-linked glycan-dependent epitope of the archetypal neutralizing antibody 2G12 is prone to evolve to alternate glycans, as revealed in this study by the negative interactions involving N295 or N332. It is imperative, therefore, to identify vulnerabilities in the form of functional constraints that limit the evolution of N-linked glycosylation sites in HIV-1 gp120. Ultimately, the characterization of constraints in the glycan shield could enable us to drive an evolving population of HIV-1 into a corner.

Although our phylogenetic and graphical models make very different assumptions about the evolution of PNGSs, we find that these complementary approaches consistently identified a set of interactions among N-linked glycosylation sites in HIV-1 envelope glycoprotein sequences. Furthermore, the results from both methods support the hypothesis that PNGSs that are located closer together in the glycoprotein tend to be mutually exclusive, and that positive interactions tend to occur between PNGSs that are more remote. Not only is this pattern intuitively appealing, but it may also represent a new and all-inclusive constraint on the evolution of PNGSs in the HIV-1 envelope sequence. Specifically, the tendency for negative interactions to occur primarily between spatially localized PNGSs implies that many N-linked glycans of divergent HIV-1 envelope glycoproteins are constrained to appear in very similar locations. Furthermore, any analysis on the frequencies of individual PNGSs in an alignment—especially those relating such quantities to a viral phenotype such as neutralization sensitivity—should count overlapping motifs as being effectively the same PNGS, because these pairs tend to manifest strong negative interactions that imply structural or functional redundancy.

## Materials and Methods

### Phylogeny reconstruction.

We obtained an alignment of published full-length HIV-1 envelope sequences from the Los Alamos National Laboratory HIV database (http://hiv-web.lanl.gov/content/hiv-db/ALIGN_04/ALIGN-INDEX.html). This alignment comprised 711 sequences, including 660 sequences representing all HIV-1 main (M) group subtypes, 44 sequences from group O, two sequences from group N, and four sequences from chimpanzee isolates of SIV. Conditional on the availability of patient or isolate annotation, this alignment had been prescreened for redundant sequences so that nearly every sequence represented a unique individual [44].

PNGSs were identified in the amino acid sequences by the occurrence of a sequence motif (i.e., sequon) NX_1_(S/T)X_2_, where X corresponds to any amino acid other than proline [2]. The position-specific frequencies of PNGSs in the alignment are reported in Figure S1. Any number of gaps in an aligned sequence was permitted to occur between residues within this motif. Amino acid sequence motifs defining overlapping PNGSs (e.g., NNSS) were counted as two distinct occurrences. Codon positions that defined a PNGS in at least one sequence were removed from the nucleotide alignment, which was subsequently used to reconstruct the phylogeny by neighbor-joining [45] using the Tamura-Nei [46] distance measure. Nonparametric bootstrap support values for the branches of this tree are reported in Figure S2.

### Phylogenetic analyses.

The HIV-1 envelope amino acid alignment was converted into binary sequences indicating the presence (1) or absence (0) of an asparagine (N) residue that initiated a PNGS at that position. We filtered all columns from the binary alignment in which no 1 entries occurred, and applied the remaining columns to the covarion and disequon model analyses that were implemented using the *HyPhy* batch language (available from http://www.hyphy.org/pubs/PNGS) [47].

The covarion model evaluated variation over time in the site-specific rates that PNGSs were gained or lost, i.e., *r*_1→0_ and *r*_0→1_, respectively. Each position in the binary alignment was permitted to switch between two hidden states, such that one state was on (*r*_0→1_, *r*_1→0_ > 0) and the second was off (*r*_0→1_, *r*_1→0_ > 0). Switches between these hidden states occurred at instantaneous rates *s*_on_ and *s*_off_. Both sets of rates were allowed to vary among positions according to factors drawn from discretized one-parameter gamma distributions. Further details on the covarion model and the constrained alternative models are provided in Protocol S1.

**Figure 4:**
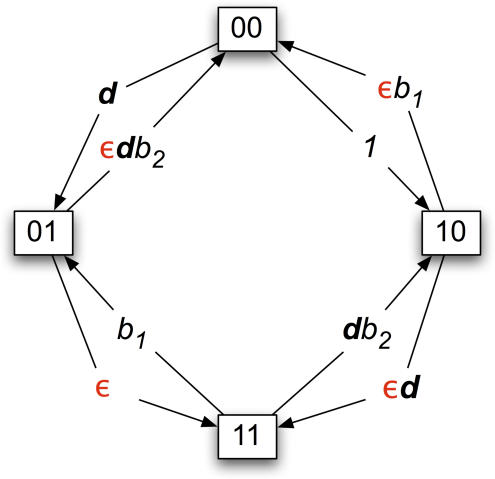
Disequon Model Parameters This diagram illustrates the eight rates that are parameterized by the disequon (paired-character) model. Each box corresponds to a state in the disequon model, in which the first digit represents the presence or absence of a PNGS at the first site, and the second digit for the second site. The interaction parameter (ɛ) between paired sites is highlighted in red.

For the disequon model analysis, we generated all pairwise combinations of columns, resulting in four possible binary paired-character states: {00, 01, 10, 11}. Simultaneous substitutions at both sites were assumed to occur at a negligibly low rate, leaving eight possible substitution rates to parameterize. The full disequon model depended on four parameters (Figure 4): *b*_1_ and *b*_2_, which quantified the asymmetry in the rate of loss over gain at the respective sites (e.g., *b*_1_ = *r*_1•→0•_/*r*_0•→1•_); the difference (*d*) in the overall evolutionary rate between the first and second sites; and an interaction parameter (ɛ). When ɛ > 1, the gain of a PNGS at one site elevated the rate of gain at the other site, and the loss of a PNGS elevated the rate of loss at the other site. Conversely, when ɛ < 1, the gain of a PNGS at one site elevated the rate of loss at the other site, and vice versa. We also evaluated a version of this model with an additional interaction parameter acting specifically on the rates *r*_01→11_ and *r*_10→11_, decoupling effects of interactions on the coupled gain and loss of PNGSs. However, the additional parameter produced no significant improvement of likelihood and we proceeded with the simpler model.

The disequon model was hence represented by the following time-reversible rate matrix:

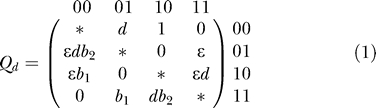
in which the rows list substitution rates from their respective states to the state indicated above each column, and the diagonal elements (*) assumed values such that each row summed to 0. Note that estimation of the rate *r*_00→10_ is confounded by branch length estimates; hence, it was fixed at 1. The stationary distribution for this model is given by π*_d_* ∝ {*b*_1_ *b*_2_ɛ, *b*_1_, *b*_2_, ɛ}, normalized to sum to 1. We fitted the disequon model to each pairwise combination of PNGSs from the alignment, using branch lengths that were constrained to be proportional to the lengths from the non-PNGS tree, given a global scaling factor. Pairwise combinations in which at least one of the four states was absent were excluded from the analysis, reducing the total number of comparisons from 49,952 to 6,455 pairs and improving the stability of model inference. A pair had a significant interaction component (ɛ ≠ 1) if the likelihood of the full model was significantly greater than that of the nested model in which ɛ was constrained to 1, i.e., by evaluating the LRT against the asymptotic null χ^2^ distribution with 1 degree of freedom. We applied a Bonferroni correction to account for multiple comparisons. This procedure is far more conservative than alternative procedures such as the false discovery rate [48]. Using the false discovery rate would have also required accurate estimation of the distribution of *p*-values under the null hypothesis, which was limited by the complexity of the likelihood surface for this model.

### Bayesian network analysis.

We chose a subset of PNGS that were present in at least 20% but fewer than 80% of the sequences in the alignment. This requirement for intermediate frequencies greatly reduced the number of PNGSs, and hence the total number of possible networks, while retaining statistical power for detecting intersite dependencies. Each PNGS was represented as a discrete (binary state) variable indicating the presence or absence of the N-linked glycosylation sequence motif at a given position in an amino acid sequence. Context-dependence between PNGSs were represented by directed arcs in a Bayesian network, so that an arc originating from *A* and terminating at *B* indicated that the probability of finding a PNGS at position *B* was conditionally dependent on whether or not a PNGS occurred at position *A*. Likewise, the absence of a directed arc indicated conditional independence between PNGSs. Our objective was therefore to estimate the joint probability distribution (encoded by the structure of directed arcs in the Bayesian network) that provided the best explanation for the distribution of PNGSs in the alignment of HIV-1 envelope sequences.

To infer, or “learn,” the structure of the network from the data, we used a parallel implementation of a greedy heuristic search algorithm with random restarts in Java [49]. We used a heuristic search algorithm because it was computationally infeasible to iterate over all possible networks (≈6.3 × 10^52^ possible networks for 17 PNGSs). Our greedy heuristic search algorithm was based on the K2 algorithm, which assumed that the dataset contained no missing values and that the prior probability distributions were uniform (i.e., such that the conditional probability *P*(*A* = 1 | *B* = 1) was equally likely to assume any real value within the interval [0,1]) [50]. Each search was initialized with a randomized network assembled by applying 100 modifications (i.e., addition, removal, or reversal of a directed arc) to an unconnected set of nodes, provided that the graph remained acyclic. Subsequently, the search algorithm iteratively evaluated the relative improvement in the posterior probability of the network from the addition, removal, or reversal of a directed arc to the current network structure, and the best modification was incorporated into the network. This iterative process continued until no further improvement in posterior probability was possible.

We applied this search algorithm to 100 random samples of 200 sequences from the alignment to evaluate the variation in the optimal network structure among sequences. The greedy search algorithm was applied to each sample with 250 random restarts to explore multiple local maxima in the scoring metric (i.e., posterior probability). Networks evaluated by the search algorithm were restricted to the subset of all possible networks in which each node had a maximum number of five parents. The restriction of the number of parent nodes to five or fewer per node was necessary to reduce the size of the search space. However, none of the locally optimal models found by our search algorithm included nodes with five parents, suggesting that the data favored simpler models. The number of occurrences of each arc, irrespective of its direction, was tabulated across all networks obtained by the search algorithm for every sample. From this table, we generated a majority-rule consensus network into which an undirected arc between the nodes *A* and *B* was incorporated if the sum of tabulated frequencies of the directed arcs *A → B* and *B → A* exceeded a threshold value of 50%. Posterior odds-ratios for each arc in the consensus network were calculated from 2 × 2 contingency tables of posterior parameters. For this calculation, undirected arcs in the consensus network were assigned directionality according to which directed arc had been sampled at a greater frequency.

Undirected arcs in the consensus network were classified as representing either positive (inclusive) or negative (exclusive) associations between PNGS, as determined by calculating the posterior odds-ratios from the corresponding 2 × 2 contingency tables of posterior parameters. For example, the contingency table for the PNGSs N411 and N413 contained the following:

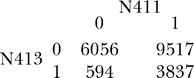
from which the posterior-odds ratio was calculated as:

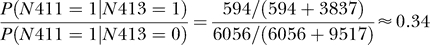
indicating that N411 was only one-third as likely to occur in a sequence that also contained a PNGS at position N413. Hence, an odds-ratio greater than 1 implied that the PNGSs were inclusive, whereas an odds-ratio less than 1 implied that they were mutually exclusive.

Because of the limited number of sequences within the HIV-1 subtypes, our analysis of subtype-specific networks was carried out using an MCMC-based procedure that is designed for inferring networks from relatively small datasets [51]. When the amount of data is small relative to the number of network variables, an exceedingly large number of networks may explain the data equally well. Rather than attempting to find an optimal network, this procedure estimates the overall posterior probability for the presence of each arc given the data. To traverse the enormous network search space more efficiently, a hierarchical order of network variables is proposed at each step, corresponding to a subset of networks in which every node is preceded by its parent in the ordered list [51]. We applied this procedure with a coupled Metropolis-Hasting sampling algorithm with at least four chains [52] to analyze subtype data.

### Visualization of protein structure.

We obtained structural coordinates for the HIV-1 glycoprotein gp120 in complex with a CD4 receptor from the Research Collaboratory for Structural Bioinformatics [33]. The location of PNGSs in the gp120 structure was visualized using the software package UCSF Chimera [53]. Note that several of the gp120 variable loops are absent from this structural prediction.

## Supporting Information

Figure S1 Frequency of PNGSs per Codon Position in HIV-1 Envelope Sequences Codon positions are numbered according to the alignment of HIV-1 envelope sequences. The approximate locations of variable loop regions and the transition between the gp120 and gp41 glycoprotein-coding domains are marked for reference. The frequency of PNGSs is normalized by the total number of sequences in the alignment (PNGSs fraction).
(35 KB PDF)

Figure S2 Bootstrap Tree of HIV-1 Envelope Sequences Internal branches are colored red to indicate >70% bootstrap support, green to indicate 50%–70% bootstrap support, and black otherwise. Bootstrap support values were estimated from 100 replicate samples. Branch lengths are derived from the neighbor-joining tree [45] based on the Tamura-Nei [46] distance measure, applied to the nucleotide alignment excluding positions at which PNGSs are defined.
(43 KB PDF)

Protocol S1 Formulation of Covarion Model of PNGSs Evolution (58 KB DOC)

Table S1 Goodness-of-Fit and Parameter Estimates for Covarion and Nested Models (81 KB PDF)

Table S2 Position and Frequency of Polymorphic PNGSs Applied to Bayesian Networks (37 KB DOC)

### Accession Numbers

The accession numbers used in this paper are from Genbank (http://www.ncbi.nlm.nih.gov/Genbank) for [NNTSNNTSY outlier] subtype B sequence (M17451) and from Protein Databank (http://www.rcsb.org/pdb/home/home.do) for folded CD4-bound gp120 protein (1G9M) and for HIV-1 glycoprotein gp120 (1G9M).

## Abbreviations

LRT: likelihood ratio test
MCMC: Markov Chain Monte Carlo
N: asparagine
PNGS: potential N-linked glycosylation site
SIV: simian immunodeficiency virus
