# Supplementary material for: Evolutionary Interactions between N-Linked Glycosylation Sites in the HIV-1 Envelope
Source: PLoS Comput Biol. 2007 Jan 19;3(1):e11. doi: 10.1371/journal.pcbi.0030011 (PMC1779302; doi:10.1371/journal.pcbi.0030011)
Supplement: Supplementary file 1 [file pcbi.0030011.sg001.pdf]

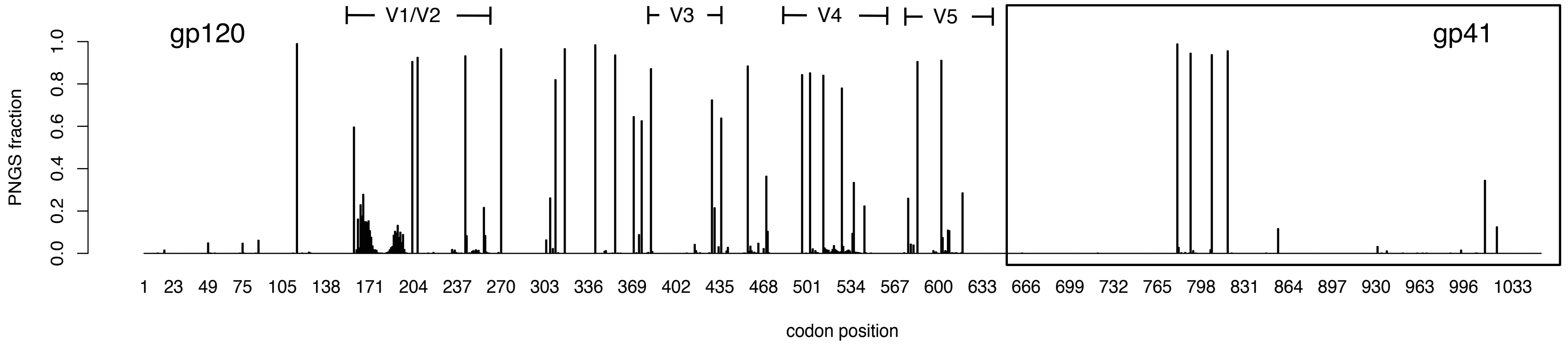

V1/V2
V3
V4
V5
gp120
gp41
PNGS fraction
0.0
0.2
0.4
0.6
0.8
1.0
1
23
49
75
105
138
171
204
237
270
303
336
369
402
435
468
501
534
567
600
633
666
699
732
765
798
831
864
897
930
963
996
1033
codon position
