# Supplementary material for: Evolutionary Interactions between N-Linked Glycosylation Sites in the HIV-1 Envelope
Source: PLoS Comput Biol. 2007 Jan 19;3(1):e11. doi: 10.1371/journal.pcbi.0030011 (PMC1779302; doi:10.1371/journal.pcbi.0030011)

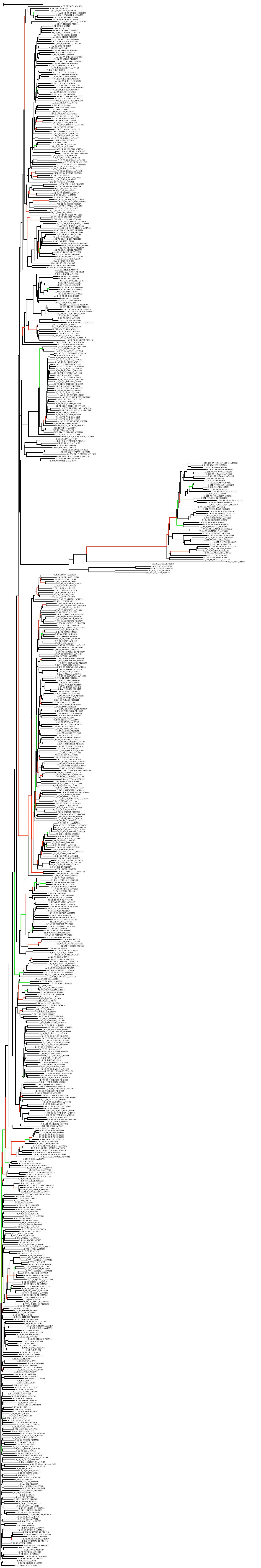

Supplement: Supplementary file 2 [file pcbi.0030011.sg002.pdf]
