# Supplementary material for: Evolutionary Interactions between N-Linked Glycosylation Sites in the HIV-1 Envelope
Source: PLoS Comput Biol. 2007 Jan 19;3(1):e11. doi: 10.1371/journal.pcbi.0030011 (PMC1779302; doi:10.1371/journal.pcbi.0030011)
Supplement: Supplementary file 3 [file pcbi.0030011.st001.pdf]

Model	df	log L	AIC	AIC$_c$	BIC	$P - value$	s_{on}	s_{off}	r_{01}/r_{10}	σ	α
1. Full model	1424	-19960.7	**42769.4**	**48453.4**	**49272.3**		0.079	0.064	0.286	0.749	0.461
2. $s_{off} = s_{on}$	1423	-19964.0	42774.0	48458.0	49272.4	0.01 (1,2)	0.076	(0.076)	0.289	0.742	0.469
3. $1/\sigma = 0$	1423	-20047.8	42941.6	48625.6	49440.0	$\ll$ 0.001 (1,3)	0.054	0.036	0.297	NA	0.472
4. $r_{01} = r_{10}$	1423	-20203.9	43253.9	48937.9	49752.2	$\ll$ 0.001 (1,4)	0.063	0.087	(1)	0.841	0.395
5. $s_{off} = 0$	1422	-20367.7	43579.5	49263.5	50073.2	$\ll$ 0.001 (3,5)	NA	(0)	0.144	NA	0.536
6. $1/\alpha = 0$	1423	-20444.9	43735.8	49419.8	50234.2	$\ll$ 0.001 (1,6)	0.044	0.121	0.295	0.580	NA

Parameter definitions: df = degrees of freedom; L = likelihood; AIC = Akaike's Information Criterion; AIC$_c$ = small-sample corrected AIC; BIC = Bayesian information criterion; s_{on}, s_{off} = rates of switching to hidden states 'on' and 'off', respectively; r_{01}, r_{10} = rates of potential N-linked glycosylation site (PNGS) gain and loss, respectively; σ = shape parameter for gamma-distributed rate variation in s_{on} and s_{off} across sites; α = shape parameter for gamma-distributed rate variation in r_{01} and r_{10} across sites. P-values were estimated by a likelihood ratio test between the nested models indicated by numbering within parentheses. Minimized AIC, AIC$_c$, and BIC values are shown in boldface to indicate that the corresponding model is favored by the criterion.

Table S1.
Goodness-of-fit and parameter estimates for covarion and nested models.
