# Supplementary material for: Evolutionary Interactions between N-Linked Glycosylation Sites in the HIV-1 Envelope
Source: PLoS Comput Biol. 2007 Jan 19;3(1):e11. doi: 10.1371/journal.pcbi.0030011 (PMC1779302; doi:10.1371/journal.pcbi.0030011)
Supplement: Supplementary file 4 [file pcbi.0030011.sd001.doc]

Evolutionary interactions between N-linked

glycosylation sites in the HIV-1 envelope

Art F.Y. Poon, Fraser I. Lewis, Sergei L. Kosakovsky Pond, and

Simon D.W. Frost

Department of Pathology, University of California, San Diego

9500 Gilman Drive, La Jolla, CA 92093, U.S.A.

Institute of Evolutionary Biology, University of Edinburgh

West Mains Road, Edinburgh EH9 3JT, Scotland, U.K.

**Protocol S1.**

We evaluated a hierarchy of increasingly complex models allowing for variation across sites in the rates of gain or loss of PNGS (*r*_0→1_ and *r*_1→0_) and the rates of switching between hidden states (*s*_on_ and *s*_off_). Variation across sites in *r*_0→1_ and *r*_1→0_ was modeled by four rate categories (, *i* = {1, 2, 3, 4}) obtained from the means of four equally-probable intervals within a one-parameter gamma distribution, which has variance of 1 / α and unit mean. To allow variation in hidden-state switching rates across sites, *s*_on_ and *s*_off_ were modified by a factor drawn from one of four rate categories (, *i* = {1, 2, 3, 4}) with values obtained from the means of four equally-probable intervals determined by a one-parameter gamma distribution with variance 1 / σ and unit mean. The full covarion model was therefore defined by the following time-reversible rate matrix:

(2)

where the rows list substitution rates from their respective states to the state indicated above each column, and the diagonal elements (‘*’) assume values such that each row sums to 0. This process has the stationary distribution π_c_ = {π_1_π_on_, π_0_π_on_, π_1_π_off_, π_0_π_off_}, where π_1_ = *r*_0→1_ / (*r*_1→0_ + *r*_0→1_), π_0_ = 1 – π_1_, π_on_ = *s*_on_ / (*s*_on_ + *s*_off_), and π_off_ = 1 – π_on_ [27]. Substitution probabilities were obtained by taking the matrix exponential of *Q*_c_*t*, where *t* is the branch length estimated for the phylogeny of the non-PNGS nucleotide partition of the alignment. We evaluated the improvement in goodness-of-fit between nested models of evolution by the significance of the likelihood ratio test statistic (LRT) compared to the asymptotic null χ^2^ distribution with the appropriate degrees of freedom (Table S1). Because the non-covarion model was constrained to remain in the ‘on’ hidden state (*s*_off_ = 0), variation among sites in the rate of switching between hidden states (quantified by 1/σ) became a non-identifiable parameter. Accordingly, we compared the performance of the non-covarion model to the covarion model without variation in switching rates (1 / σ = 0, *i.e.* *S*_i_ = 1 for all *i*).
