# Supplementary material for: Evolutionary Interactions between N-Linked Glycosylation Sites in the HIV-1 Envelope
Source: PLoS Comput Biol. 2007 Jan 19;3(1):e11. doi: 10.1371/journal.pcbi.0030011 (PMC1779302; doi:10.1371/journal.pcbi.0030011)
Supplement: Supplementary file 5 [file pcbi.0030011.st002.doc]

**Table S2.**

Position and frequency of polymorphic PNGS applied to Bayesian network.

| codon position | | region | frequency |
| --- | --- | --- | --- |
| alignment | HxB2 |  | |
| 159 | 130 | V1 | 0.60 |
| 164 | 134 | V1 | 0.23 |
| 166 | 136 | V1 | 0.28 |
| 257 | *V2* | V2 | 0.22 |
| 307 | 230 | C2 | 0.26 |
| 370 | 289 | C2 | 0.65 |
| 376 | 295 | C2/V3 | 0.63 |
| 429 | 332 | C3 | 0.72 |
| 431 | 334 | C3 | 0.22 |
| 436 | 339 | C3 | 0.64 |
| 470 | 362 | C3 | 0.36 |
| 527 | 406 | V4 | 0.78 |
| 536 | 411 | V4 | 0.34 |
| 544 | 413 | V4 | 0.22 |
| 577 | 442 | C4 | 0.26 |
| 618 | 465 | V5 | 0.29 |
| 1012 | 816 | gp41 | 0.34 |
